# NEMO recruitment at single cytokine-receptor complexes shows quantized dynamics independent of ligand affinity

**DOI:** 10.1016/j.celrep.2025.116637

**Published:** 2025-12-02

**Authors:** A. Hyun Kim, Benjamin Krummenacher, Jason Yeung, David R. Koes, Robin E.C. Lee

**Affiliations:** 1 Department of Computational and Systems Biology, School of Medicine, University of Pittsburgh, Pittsburgh, PA 15213, USA; 2 Center for Systems Immunology, School of Medicine, University of Pittsburgh, Pittsburgh, PA 15213, USA; 3 Department of Physics and Astronomy, University of Pittsburgh, Pittsburgh, PA 15213, USA; 4 These authors contributed equally; 5 Lead contact

## Abstract

Cells use a limited number of receptors to sense and process molecular information from their environment. In the classical view of signaling, receptor-ligand affinities determine binding kinetics, on timescales of diffusion, where their time-averaged contact duration regulates rapid cytoplasmic signaling events to coordinate cellular responses. For some cytokines, single receptor-ligand binding events can initiate large multiprotein complexes in the cytoplasm that assemble over tens of minutes, raising the question of how cytokine affinity influences the sensitivity and strength of signaling. Here, we leverage naturally occurring variations of interleukin (IL)-1β from multiple species to determine the impact of affinity on human IL-1 receptor signaling. Using experiments and models, we investigate single receptor complexes activated by ligands that vary across multiple orders of magnitude in affinity. Our results show that while the receptor-ligand affinity establishes cytokine response sensitivity, activated IL-1 receptor complexes signal as discrete, quantized packets of signaling flux independent of affinity.

## In brief

Kim et al. leverage natural variation in IL-1β affinity across species to show that while ligand affinity sets activation thresholds, each IL-1 receptor signaling complex outputs a fixed, quantized burst of signal. This mechanism provides robustness and specificity to cytokine responses and further defines mechanisms of cytokine signal encoding.

## Graphical Abstract

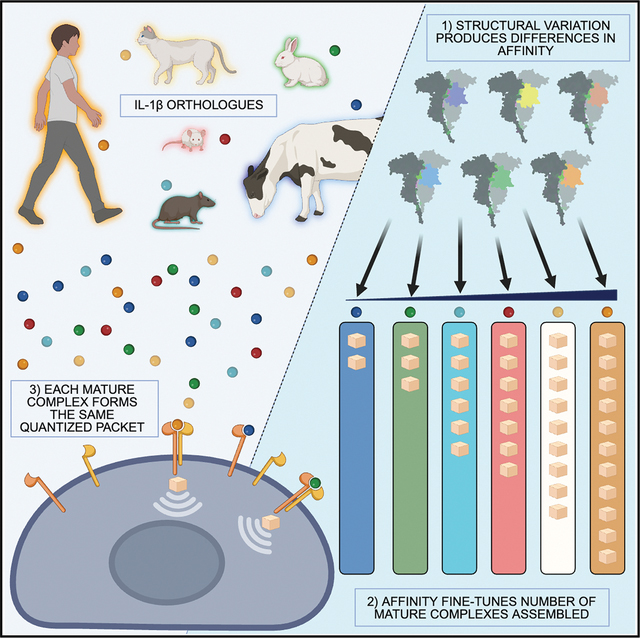

## INTRODUCTION

When extracellular ligands bind to transmembrane receptors, intracellular signaling cascades are initiated that regulate essential biological functions.^1^ Berg and Purcell’s classic work showed that the integrated time-averaged occupancy of a receptor enables cells to infer extracellular ligand concentrations,^2^ while later extensions using fluctuation-dissipation theory formalized how affinity shapes the precision of downstream responses.^3–5^ These theories establish that ligand-receptor kinetics encodes signaling outcomes, making affinity a key regulator of cell behavior.

Although originally developed for bacterial chemotaxis, these principles apply broadly to eukaryotic signaling. For ligand-gated ion channels, contact duration directly regulates channel activity,^6^ and for G-protein-coupled receptors (GPCRs), binding duration determines signaling output.^1^ Ligand binding to GPCRs activates Gα by catalyzing GDP-GTP exchange, causing dissociation from Gβγ and initiating downstream signaling.^7^ Signal termination depends both on G-protein engagement and ligand affinity.^8^ For example, the adenosine A1^9^ and dopamine D2^10,11^ GPCRs rapidly terminate signaling upon ligand dissociation, highlighting how residence time shapes cellular outcomes in disorders such as Parkinson’s and schizophrenia.

Similarly, receptor tyrosine kinases (RTKs) adjust signaling based on ligand affinity. Ligand engagement triggers dimerization and autophosphorylation, recruiting effectors such as Ras, PI3K, and STATs.^12^ Longer binding sustains phosphorylation and scaffold recruitment, amplifying MAPK and PI3K signaling, while brief interactions favor transient pathways.^13,14^ RTK internalization is likewise influenced by occupancy time. Non-catalytic tyrosine-phosphorylated receptors (NTRs), including T cell receptors, rely on kinetic proofreading to discriminate weak versus strong interactions that regulate transient versus sustained signaling responses.^15,16^

Altered affinity or binding duration frequently underpins disease. In cancer, elevated ligand affinity or receptor overexpression causes aberrant RTK activation and resistance to apoptosis.^17–19^ For instance, affinity-increasing extracellular EGFR mutations promote oncogenic transformation.^20^ In neurodegeneration, altered GPCR dynamics compromise neurotransmission: patients with Alzheimer’s show reduced ligand binding to neurotransmitter receptors, contributing to cognitive decline.^21^ These examples demonstrate how physical parameters, such as residence time, critically influence cellular responses in health and disease.

The nuclear factor κB (NF-κB) pathway exemplifies how extracellular cytokines regulate specific gene expression programs.^22–24^ NF-κB controls diverse processes, from pathogen defense to tissue homeostasis, and its dysregulation is implicated broadly in diseases.^25–29^ Under basal conditions, NF-κB is sequestered in the cytoplasm by its inhibitor, IκB.^30–32^ Canonical NF-κB signaling is initiated when cytokines such as tumor necrosis factor (TNF) or interleukin (IL)-1β bind their receptors at the plasma membrane, triggering the assembly of receptor-proximal complexes that activate the IκB kinase (IKK) complex. Activated IKK phosphorylates IκB, targeting it for proteasomal degradation, and promotes nuclear translocation of NF-κB.^33–35^

Despite using distinct receptors, cytokine signaling converges on several key steps. TNF binding to TNF receptor 1 (TNFR1) recruits the adaptor molecule TRADD and other mediators to form complex I (CI), a membrane-associated hub that nucleates nondegradative linear and branched ubiquitin chains, which in turn recruit IKK complexes. Similarly, IL-1β binding to IL-1 receptor 1 (IL-1R1) and its co-receptor IL-1R3 recruits MyD88 and other molecules to form a CI-like structure^36^ that also anchors polyubiquitin to coordinate IKK activity by induced proximity.^37–39^ Collectively, CI-like complexes regulate multi-step feedback loops that determine signal amplitude and duration.^40,41^ The formation of dynamic supramolecular complexes distinguishes this system from many receptor classes.

A natural question arises: how do cytokine-receptor affinity and contact duration impact downstream signals? CI-like complexes from the same cytokine exhibit remarkable similarity in their dynamics across concentrations,^40^ hinting at a possible departure from classical receptor signaling. Here, we probe the affinity space of IL-1β to determine its effect on single activated receptor complexes. Using phylogenetic and structural analyses of IL-1β homologs, we infer that orthologs from different species will bind to human receptors with altered kinetics. We validate this in human cells expressing a CI-like reporter and observe shifted dose-response curves where affinity alters potency. From computational models and single-particle tracking (SPT), we show that despite several orders of magnitude variation in inferred affinity, the signal strength and dynamics of single CI-like complexes are invariant. Thus, while affinity fine-tunes activation thresholds, each activated CI-like complex produces a quantized and affinity-independent signaling flux in the cell.

## RESULTS

### Naturally occurring sequence and structure variation in IL-1β is predicted to alter affinity for IL-1R1

**Figure 1.**
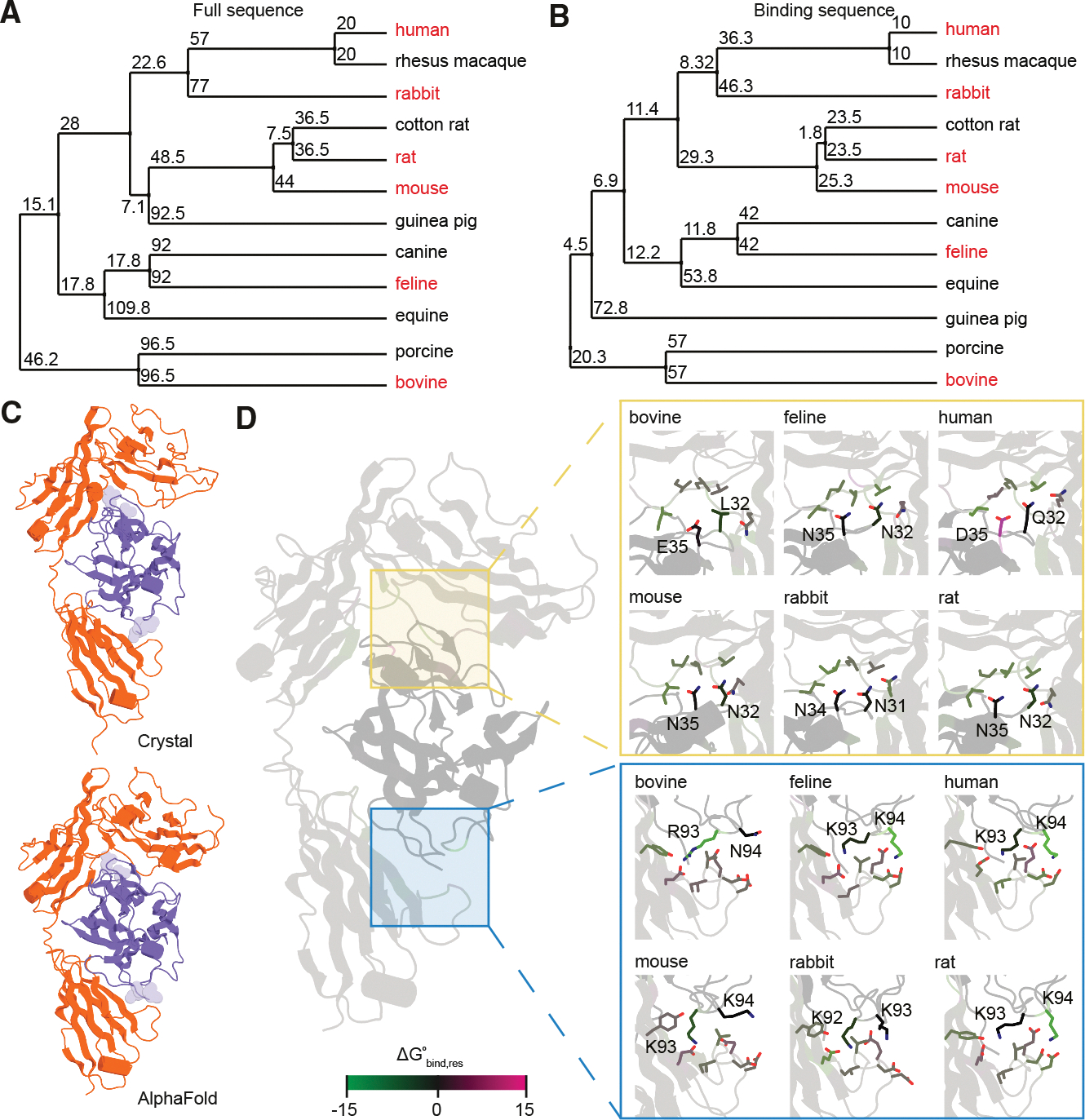
Cross-species comparison of IL-1β sequence and structure (A) Average distance trees of IL-1β full sequences across divergent species. (B) Average distance trees across predicted receptor-ligand binding regions. The red font indicates species selected for subsequent analysis. (C) Crystal and generated structure for IL-1β (blue) in complex with the extracellular domain of IL-1R1 (orange). (D) Differences in binding conformations across generated structures of IL-1β-IL-1R1 complexes for two predicted binding interfaces, with residues colored by their estimated contributions to the change in free energy. See also Figure S1.

To probe the molecular basis of IL-1R1-dependent signal transmission, we performed a cross-species multiple sequence alignment of IL-1β. Using a curated panel of mammalian species spanning over 100 million years of evolutionary divergence, we compared full-length IL-1β sequences and constructed a distance tree based on sequence dissimilarity (Figure 1A). This revealed species-specific variation, stratified by evolutionary proximity; for example, rodents’ IL-1β clustered separately from primate or ungulate sequences.

We next restricted our comparison to the IL-1R1-binding interface from the human IL-1β-IL-1R1 co-crystal structure (PDB: 1ITB^42^). We identified receptor-contacting residues and extracted the corresponding positions from each species’ sequence (Figure S1A). The distance tree based on contact residues paralleled the full sequence, suggesting that binding interface variation tracks with overall evolutionary divergence (Figure 1B). These substitutions at the receptor-ligand interface could modulate affinity and, by extension, downstream signaling responses in the cell. Evolutionary drift in IL-1β’s binding interface may therefore underpin receptor-ligand co-evolution or species-specific fine-tuning for aspects of signal transduction.^43,44^

Next, we simulated the molecular dynamics (MD) of predicted chimeric IL-1β-IL-1R1 complexes. AlphaFold3 predictions of the human IL-1β-IL-1R1 complex closely recapitulated the experimental co-crystal,^8^ validating the approach (Figures 1C and S1B). Then, for 5 representative species spanning the distance tree, we generated IL-1β orthologs bound to human IL-1R1. Binding interface analysis revealed varying similarity to the human complex, particularly in loop regions implicated in receptor contact (Figure 1D). Short simulations estimated changes in binding free energy (ΔG°) for each chimera using molecular mechanics/generalized Born surface area (MM/GBSA).^45^ Differences in ΔG° values spanned nearly two orders of magnitude across species (Figure S1C). Single-residue contributions of ΔG° were determined using the MM/Poisson-Boltzmann surface area (MM/PBSA) method.^45^ Regions of low ΔG° aligned with binding interfaces, again suggesting local variations that alter affinity (Figures 1 and S1).

We also examined the tripartite complex formed with the accessory co-receptor IL-1R3.^46,47^ AlphaFold3 predictions of the human IL-1β-IL-1R1-IL-1R3 structure closely recapitulate the experimental co-crystals (PDB: 4DEP^46^), preserving local and global conformations (Figure S1B). Taking the same panel of IL-1β orthologs, we performed MD simulations and calculated the ΔG° of the tripartite complex, which largely mirrored the stratification of the bipartite complexes (Figure S1C). Residuelevel comparisons of ΔG° calculated using MM/PBSA^45^ revealed sequence-dependent differences in local interaction favorability between IL-1R3 and each IL-1β ortholog (Figure S1B).

Consequently, we asked whether differences at the tripartite C termini would alter cross-membrane MyD88 engagement. Assuming approximately rigid transmembrane domains, the distance between the C-terminal tails of IL-1R1 and IL-1R3 provides a proxy for Toll/IL-1R (TIR) domain separation, a determinant of MyD88 recruitment. In the crystal structure, this distance is 19.9 Å,^46^ which we used to predict competent signaling. Across simulations, the distance between the C termini remained near this value (Figure S1D), suggesting that all ortholog complexes can form signaling-competent CI-like assemblies. Interestingly, human complexes showed the largest variance in TIR spacing, raising the argument that increased flexibility may influence MyD88 binding.

Predicted ΔG° values provide relative binding strengths, which correspond to affinity. Bovine IL-1β orthologs showed consistently higher predicted ΔG° (weaker affinity) compared to most other species and especially so for felines (strongest affinity). The predicted affinity for human IL-1β was weaker than expected, likely due to the generated conformation of Asp35, showing an approximately 4-fold increase in ΔG° relative to the crystal structure. However, this does not preclude the possibility of allostery for human IL-1β-IL-1R1 that facilitates other favorable interactions in the multiprotein complex, despite reduced predicted affinity. Taken together, these findings highlight how natural sequence variation shapes both binding energy and complex geometry. Naturally occurring variations in IL-1β from felines to bovine give rise to a spectrum of ligand-receptor conformations, all of which may produce competent CI-like assemblies based on similarity of their tripartite structures.

### CI-like assemblies observed in living cells reveal species-specific response sensitivities

U2OS expressing an endogenous fusion of EGFP-NEMO provides two outputs^40,41^: first, over timescales of hours, it informs when signaling-competent CI-like receptor complexes form and dissipate based on the binary detection of NEMO assemblies; second, from time courses of NEMO intensity at single assemblies on the timescales of seconds to minutes, cytokine-specific kinetics of NEMO recruitment and dissolution at each CI-like complex can be measured. Together, these observables at the plasma membrane are predictive of the sensitivity, timing, and strength of downstream NF-κB pathway activation in the same cell.^40,41^

**Figure 2.**
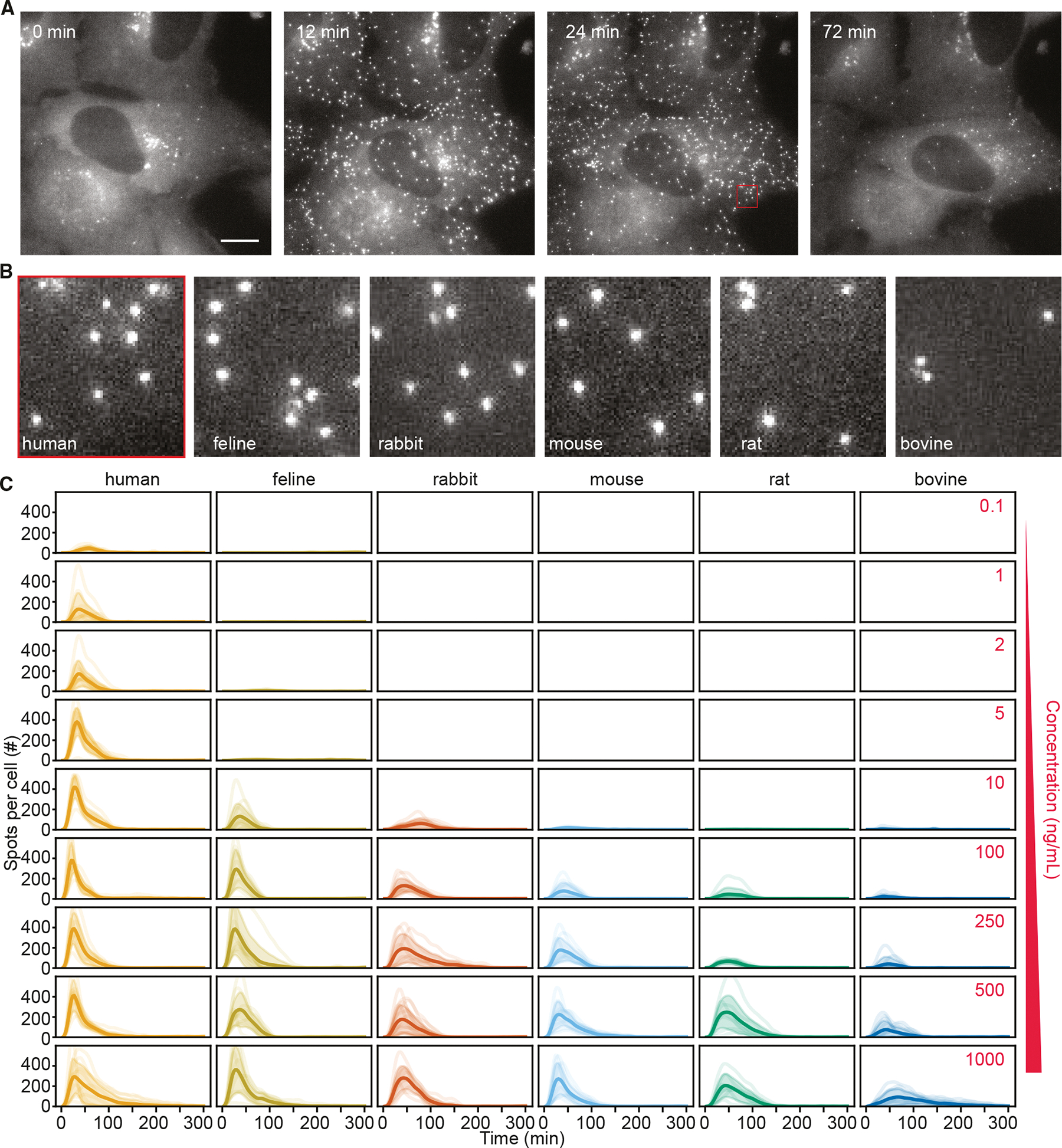
EGFP-NEMO assemblies form with varying sensitivities to IL-1β from different species (A) Time-lapse images of EGFP-NEMO endogenously expressed in U2OS cells stimulated with 250 ng/mL human IL-1β. Scale bar, 20 μm. (B) Magnification of EGFP-NEMO assemblies following 24 min of stimulation with 250 ng/mL IL-1β from indicated species. For scale, the red inset box is displayed in (A). See also Video S1 and Figure S2. (C) Single-cell time courses for the number of EGFP-NEMO complexes in response to indicated concentrations and species of IL-1β. See also Figure S2.

We examined single-cell dynamics of EGFP-NEMO using live-cell time-lapse microscopy following stimulation with recombinant IL-1β from the same panel of species (Figure 2; Video S1). These cytoplasmic assemblies represent organizing hubs of the IKK complex that regulate its activation,^40,41,48^ enabling us to measure signaling competency of cross-species cytokine stimulation (Figures 2A and S2A). Remarkably, EGFP-NEMO at mature CI-like assemblies across all species was similar in appearance and intensity (Figures 2B and S2B), suggesting a conserved molecular composition once complex formation is initiated.

We measured EGFP-NEMO puncta across IL-1β concentrations spanning four orders of magnitude and extracted a single-cell time course (Figure 2C). As shown previously, EGFP-NEMO puncta numbers peaked within 30 min and adapted back to baseline values within 60–120 min. While all orthologs showed stimulus-dependent puncta formation, the amplitude and duration of the response varied considerably between species, with humans showing the greatest sensitivity to lower concentrations, followed by felines, and then rabbits and rodents. The sensitivity of the response to bovine IL-1β was markedly weaker.

To confirm this pattern, we generated an endogenous EGFP-NEMO fusion in A549 cells using CRISPR-Cas9 (Figure S2C; Video S2). Stimulation with the selected orthologs revealed similar species-dependent variations (Figure S2D). The rank order of sensitivities from both cell lines to IL-1β orthologs shows the same general pattern as ΔG° predictions (Figure S1C). Thus, all orthologs can produce signaling-competent CI-like complexes with activation thresholds consistent with their respective predicted affinities.

### Experiments and models show that species-specific ligand affinities span over log decades

**Figure 3.**
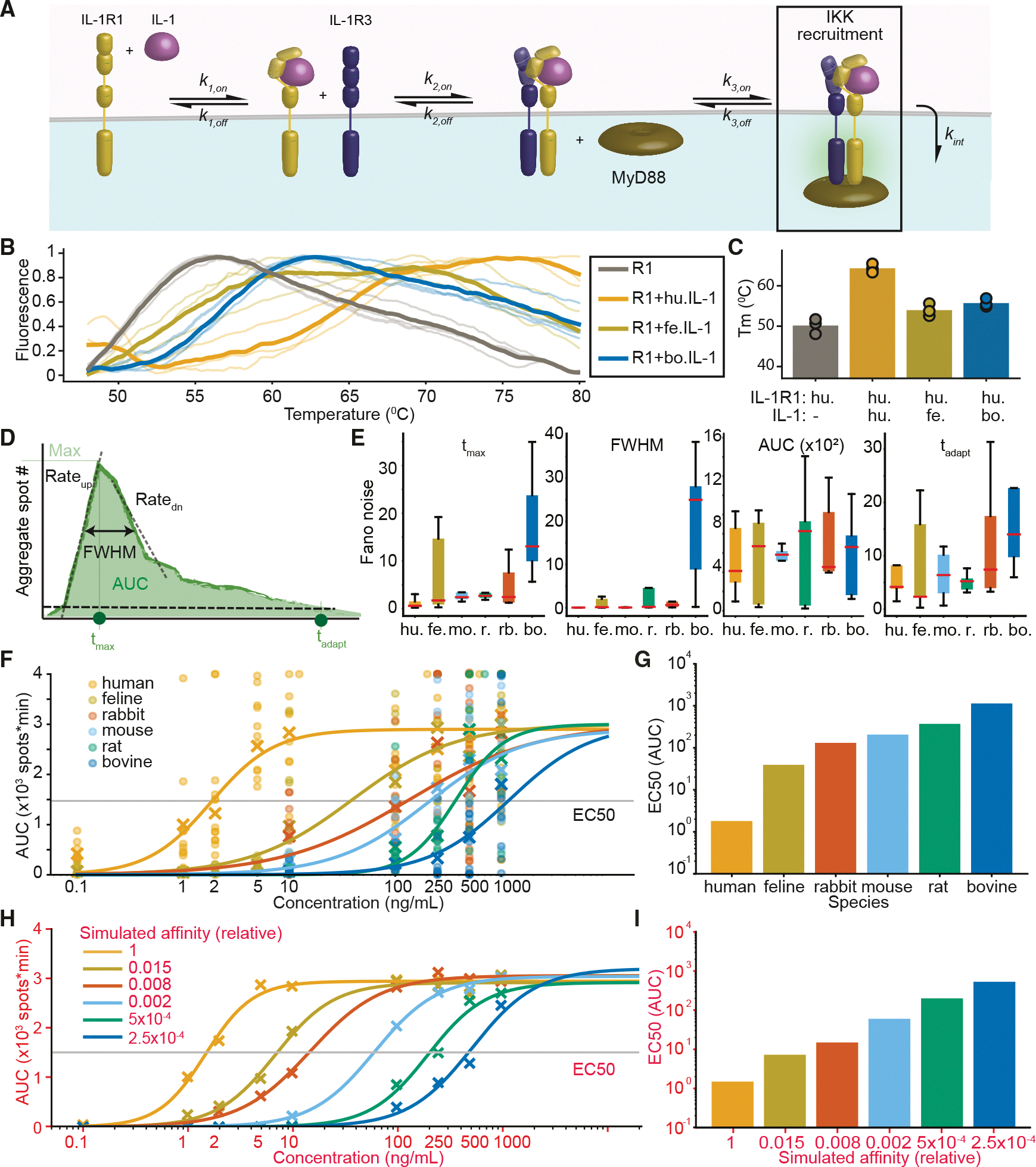
Dose responses of EGFP-NEMO dynamics reveal the predicted range of cross-species net affinity for CI-like assemblies (A) Schematic of IL-1β-induced EGFP-NEMO complex formation at the plasma membrane. IL-1β first binds to IL-1R1, enabling recruitment of IL-1R3 to form the receptor complex. Cytoplasmic MyD88 associates with the complex, facilitating IKK recruitment and the formation of EGFP-NEMO puncta. (B) Thermal shift curves of IL-1R1 stabilized by IL-1β indicate thermal stabilization of the human receptor by indicated cytokine orthologs. (C) Melting temperatures of isolated IL-1R1 as well as IL-1R1 in complex with IL-1β orthologs, derived from thermal shift curves in (B). (D) Quantitative descriptors extracted from each single-cell time courses of EGFP-NEMO puncta. (E) Boxplots of Fano noise evaluated for single-cell time courses at each concentration for indicated species. (F) Sigmoid curves fitted to the mean values of experimental single-cell descriptors across IL-1β concentrations and species. The EC_50_ is indicated. See Table S1 for fit parameters. (G) EC_50_ values, reflecting the concentration at which 50% of the maximal response is reached quantified from (F). (H) Stochastic simulations using a minimal model recapitulate experimental results. Simulated dose-response curves as in (D) reveal dose-response relationshipsfor each predicted affinity. See Table S1 for simulation and fit parameters. (I) EC_50_ values derived from simulated data, reflecting the predicted net affinity of IL-1β to form signaling-competent complexes. See Table S1 for fit parameters. See also Figure S3.

Mature CI-like assemblies form sequentially, starting with primary ligand-receptor binding, followed by recruitment of co-receptor IL-1R3 and cytoplasmic adapter proteins (Figure 3A). Using phycoerythrin-conjugated beads and antibodies, we quantified IL-1R1 and IL-1R3 surface receptor expression on both U2OS and A549 cell lines (Figures S2E and S2F). Flow cytometry revealed that R3 is limiting in A549 cells and R1 is limiting in U2OS cells. Based on surface receptors, both cell lines are expected to maximally produce several hundred signaling-competent complexes, while in parallel, we quantified the IL-1R2 decoy receptor, which was expressed at low to undetectable levels.

Next, we validated predicted ortholog binding affinities using thermal shift assays. Human IL-1R1 protein unfolded at 51.8°C and was stabilized to 65.6°C upon binding with human IL-1β (Figures 3B and 3C), consistent with previously reported melting temperatures (Tm) of the three IL-1R1 immunoglobulin-like domains.^49^ Ligand stabilization was lower with feline (Tm = 53.7°C) and bovine IL-1β (Tm = 56.8°C), consistent with their predicted binding affinities. Notably, IL-1R1 stabilized by feline IL-1β showed a prolonged, almost bi-phasic melt curve, suggesting that one of the IL-1R1 domains may be further stabilized by the feline ortholog. Consequently, the feline Tm may be an underestimation.

Features such as the “area under the curve” (AUC) for the number of CI-like complexes from single-cell EGFP-NEMO trajectories are strong predictors of dose-response characteristics.^40^ We therefore decomposed each single-cell trajectory into a set of quantitative descriptors, including AUC, peak amplitude (Max), response timing (T_MAX_, full width at half maximum [FWHM], and adaptation time), and rates of rise and fall (Rate_up_ and Rate_down_; Figure 3D). These features capture both the strength and shape of the response, enabling a quantitative measure of signaling competency and fidelity.

Cross-species variability in cell-to-cell differences was quantified by the Fano noise (Figure 3E), revealing that the lowest variability was for human responses and the greatest for bovine IL-1β. This result suggests that receptor engagement by cross-species cytokines is less coordinated. Descriptors for rise and fall, and the max number of spots, did not show any speciesspecific trends.

Dose responses based on the AUC and Max descriptors versus concentration were well fit by sigmoid curves, showing species-specific stratification (Figures 3F and S3A; Table S1). In conventional ligand-receptor kinetics, the concentration of half-maximal occupancy (EC50) from a dose-response curve embodies the K_D_, reflecting the binding affinity. For our system, the EC50 ligand concentration for a half-maximal response reflects the ortholog’s net affinity within the signaling-CI-like assembly (gray lines in Figures 3F and S3A). For both descriptors, EC50 values spanned nearly three orders of magnitude between human and bovine IL-1β, with other species distributed in between (Figures 3G and S3B).

To approximate how primary receptor-ligand binding affinity shapes signaling, we implemented a stochastic mechanistic model using the Gillespie method.^50^ This minimal model approximates CI-like complex assembly through a three-step reversible binding cascade: (1) IL-1β binds IL-1R1 to form the primary complex, (2) the IL-1R3 accessory protein binds to form a tripartite intermediate, and (3) MyD88 binds as a terminal adaptor to complete the complex and initiate downstream NEMO recruitment (Figure 3A; STAR Methods). The mature complex dissociates due to either degradation or internalization.^51,52^ To isolate the contribution of receptor-ligand affinity, we held all other kinetic parameters constant across species and varied only the IL-1β-IL-1R1 on and off rates. Because subsequent steps in forming the mature CI-like complex depend on ligand binding, the simulated affinity of the IL-1β-IL-1R1 interaction can be interpreted as a net affinity for the CI-like assembly. After fitting the model to human dose responses for the number of CI-like assemblies, we found that all other species could be recapitulated by scanning the net affinity of IL-1β (Figures 3H and S3C; Table S1). Extracted AUC and MAX descriptors from simulated data inferred that the net affinity of human IL-1β is 2–4 log decades stronger than feline and bovine, respectively, with rabbit and rodent interspersed between (Figures 3 and S3; Table S1). These results indicate that differences in affinity between IL-1R1 and IL-1β orthologs lead to significant changes in CI-like assembly propensity.

### Single CI-like structures show quantized NEMO recruitment regardless of affinity

**Figure 4.**
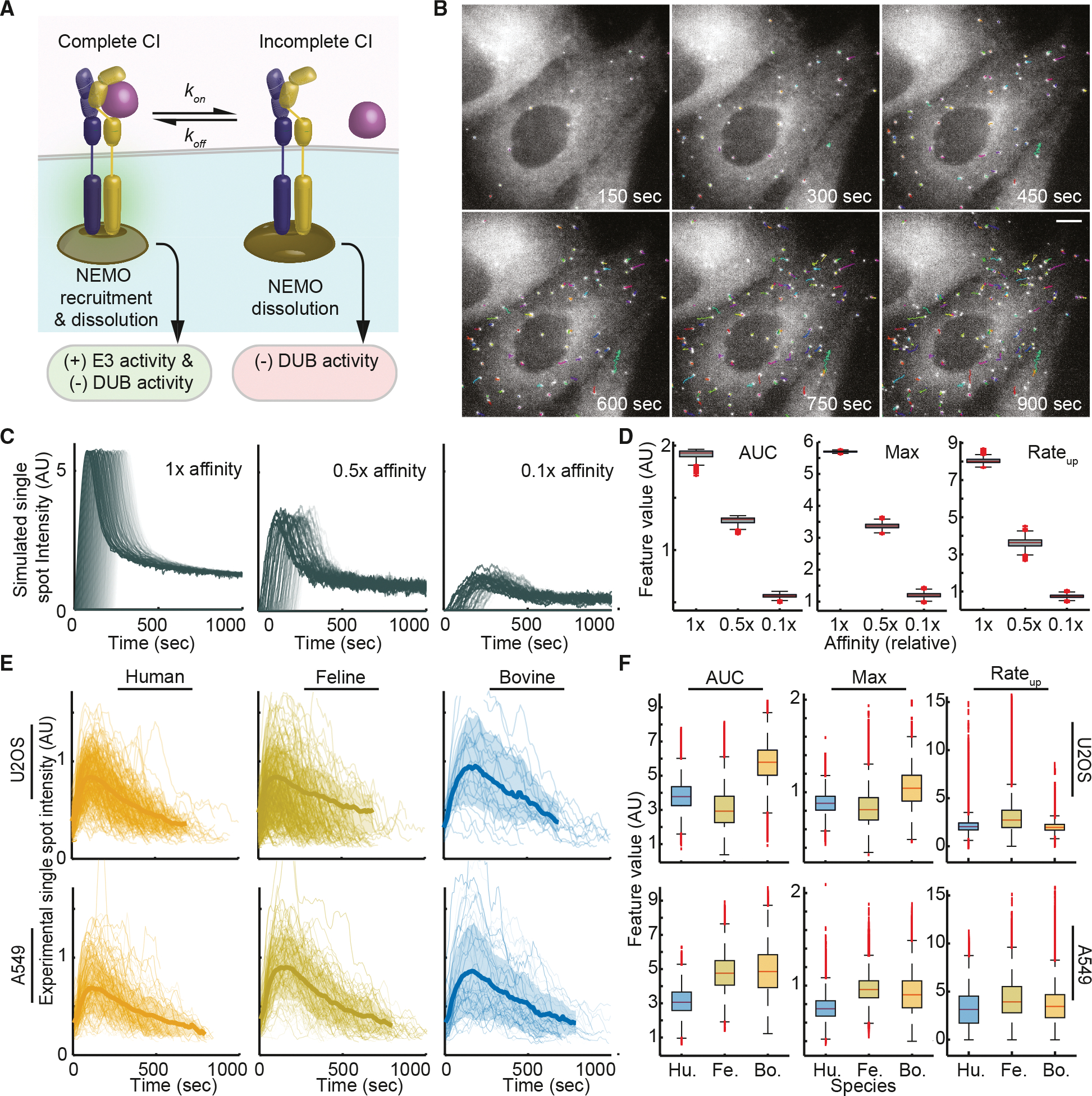
EGFP-NEMO assemblies show quantized dynamics that are independent of IL-1β affinity (A) Schematic of classical receptor signaling. In the cytokine-bound state, E3 activity promotes ubiquitin chain growth and recruitment of NEMO, while DUBscontribute to ubiquitin chain disassembly. NEMO complexes are degraded by basal DUB activity both in the presence and absence of cytokine binding. (B) Rapid time-lapse imaging shows single-particle tracks of EGFP-NEMO complexes in a single cell. Scale bar, 20 μm. See Video S3. (C) Stochastic simulations of spot-intensity time courses for single CI-like complexes, where affinity influences contact duration and consequent NEMOrecruitment. See Table S1 for parameter values. See also Figure S4 for alternative affinity-dependent mechanisms. (D) Boxplots of descriptors from simulated trajectories illustrating the effects of a one-order-of-magnitude reduction in affinity on single-complex dynamics. (E) Fast imaging and SPT of EGFP-NEMO complexes in live cells exposed to IL-1β for the indicated species and cell line. Each trajectory represents a single assembly. The average trajectory (bold line) and the standard deviation (shaded region) are also shown. (F) Boxplots analogous to (D) from experimental time courses of single complexes. Despite an expected three-orders-of-magnitude reduction for bovine affinity relative to human IL-1β, single spot trends do not show significant reductions (left-tail *t* test, *p* > 0.05 for all). See also Figure S4.

CI-like complexes can form robustly in response to a range of IL-1β orthologs despite distinct binding kinetics. From the lens of classical signaling, we expect IL-1β orthologs with lower net affinities will produce lower signaling flux into the cell. One way to invoke expectations of classic signaling to IL-1β-IL-1R1 is by considering the contact duration relative to the net affinity of the cytokine for the mature receptor complex. Here, the ligand-bound complex contributes positively to ubiquitin chain growth and stability through E3 ligases, leading to NEMO recruitment, whereas the unbound form does not (Figure 4A). Both bound and unbound forms are subject to polyubiquitin degradation through the action of deubiquitinating enzymes (DUBs), as described previously.^40,48,53^

By combining models and rapid imaging experiments coupled to SPT (Figure 4B; Video S3), we tested the impact of IL-1β net affinity on the dynamics of NEMO recruitment to CI-like assemblies. We previously described a stochastic model simulating formation and dissociation events to describe the kinetics of IKK recruitment to CI assemblies.^40^ While this framework does not capture the fully adaptive behavior seen experimentally, it offers a minimal model for simulating the growth and decay processes observed for fluorescent spots. Using the model, we first simulated the effects of contact duration on activating complex growth mechanisms. We observed that reducing the net ligand affinity (1) reduces the AUC of NEMO recruited to each simulated complex, (2) reduces the Max number of NEMO molecules per simulated complex, and (3) reduces the rate of complex formation (Figures 4C and 4D). We also simulated the effect of contact duration on the rate of ubiquitin-mediated growth and NEMO recruitment, as well as the effects of contact duration on feedforward-mediated complex degradation, with similar effects (Figure S4; Table S1). For all model variants, simulations suggest a 4- to 8-fold reduction of NEMO recruitment at CI-like structures for ligands with feline-like net affinity and much steeper reductions for ligands with bovine-like affinity.

Next, we experimentally measured intensity time courses for single EGFP-NEMO assemblies following exposure to selected orthologs (Figure 4B; Video S2). To ensure that single-particle tracks were comparable and minimally impacted by photobleaching, we only considered new spots forming within the first 4 min of imaging, pooling spots from multiple cells. Remarkably, descriptors for live-cell SPT trajectories did not show significant reductions in quantitative features (Figure 4F; left-tail *t* test, *p* > 0.05). Instead, bovine IL-1β-induced spots showed slight increases against the model-predicted trend, although these differences are likely due to the exceedingly low number of observable trajectories used for bootstrapping. Overall, trajectories of CI-like assemblies are strikingly similar when compared between different cells in both U2OS and A549 cell lines (Figure 4E) and the response of these cells to IL-1β orthologs with highly dissimilar net affinity. Taken together, our results show that single receptor complexes show a quantized behavior: once the complex matures, there is a predictable pattern of downstream signaling flux within the cell that is independent of the net affinity of IL-1β for the receptor and the mature receptor complex.

## DISCUSSION

Affinity-dependent signaling governs many rapid biological processes, yet its role in cytokine systems that operate on slower, larger scales is less clear. Our results indicate that cytokine receptor signaling deviates from classical models. Rather than scaling with ligand-receptor affinity, signaling is quantized, where each cytokine-receptor complex produces a discrete and predictable unit of signaling flux.

Quantization provides a framework for interpreting how cells encode cytokine identity. Differences in NEMO recruitment at CI-like assemblies could represent discrete packets of information that the cell decodes to elicit appropriate responses to specific extracellular challenges.^54^ This enables shared pathways to process diverse cues through temporal rather than amplitude-based encoding alone.

This view also alters concepts and sources of robustness in cytokine signaling. Mutations and perturbations that alter cytokine-receptor affinity may shift sensitivity thresholds but not the quantized output of each complex. By contrast, perturbations to cytoplasmic components, such as E3 ligases or DUBs, could alter the quantized code, leading to context-confused or ambiguous signaling. Evaluating cytokine signaling and therapeutics through the lens of quantization may therefore reveal new control points and strategies.

While quantized receptor signaling still uses affinity and contact duration to define sensitivity thresholds, it may confer advantages, including robustness, predictable amplification, and temporally encoded information transfer. Conceivably, cells could use such time-domain encoding strategies analogously to Morse code, where meaning arises from the pattern and timing of discrete events. Further characterization of the dynamics and quantization of CI-like assemblies will identify new nodes of cytokine control and illuminate underlying biological design principles.

### Limitations of the study

Two primary limitations exist. First, predicted binding free energy differences raise the possibility that IL-1β orthologs may compete for decoy receptors, such as IL-1R2.^55–57^ While our dual-reporter cell lines express negligible IL-1R2 (Figures S2E and S2F), future studies with tunable decoy receptor expression could clarify how affinity-driven receptor competition occurs. Second, MD simulations used extracellular receptor domains only. By excluding the transmembrane and intracellular domains, we may overlook constraints that influence binding energy and receptor dynamics. Also, because generative protein models recapitulate experimental structures, the AlphaFold-generated human IL-1β complexes may not represent the lowest-energy conformations. These limitations also apply to thermal shift assays. Future work incorporating full-length and transmembrane receptors with improved generative models will better link predictions with experiments.

## RESOURCE AVAILABILITY

### Lead contact

Additional information, as well as requests for resources and reagents, should be directed to and will be fulfilled by the lead contact, Robin E.C. Lee (robineclee@pitt.edu).

### Materials availability

This study did not generate new unique reagents.

### Data and code availability

- All data in this paper will be shared by the lead contact upon request.
- All original code is available in Data S1 and has been deposited on GitHub (for the Zenodo DOI, see the key resources table).
- Any additional information required to reanalyze the data reported in this paper is available from the lead contact upon request.

## STAR★METHODS

### EXPERIMENTAL MODEL AND STUDY PARTICIPANT DETAILS

U2OS cells (ATCC) endogenously expressing EGFP-NEMO and mCherry-RelA,^40,58^ as well as A549 cells (ATCC) stably expressing EGFP-NEMO, were cultured in McCoy’s 5A medium and F-12K medium (Thermo Fisher), respectively. Both were supplemented with 10% fetal bovine serum (FBS; Corning), 100 U/mL penicillin, 100 μg/mL streptomycin, and 0.2 mM L-glutamine (Invitrogen). Cells were maintained at 37°C in a humidified incubator with 5% CO_2_ and routinely tested for mycoplasma contamination using PlasmoTest Mycoplasma Detection Kit (InvivoGen).

### METHOD DETAILS

#### Establishing EGFP-NEMO knock-in cells

Reporter cell lines were engineered using CRISPR/Cas-9 technology.^40,58^ The IKBKG homology arms consisted of an 861 bp left homology arm (LHA, chrX:154551142–154552002) and a 797 bp right homology arm (RHA, chrX:154552006–154552798) flanking an EGFP coding sequence with a start codon, no stop codon, and a 3x GGSG linker. After synthesizing the sequence from GeneArt, the following primer pairs were used for amplification: IKBKG_EGFP_F 5′TCT GCT GGG TAA GGA TGT G3′, IKBKG_EGFP_R 5′GCT CTT GAT TCT CCT CCA GGC AG3′. Following purification, fragments were cloned into pMK digested with AatII by Gibson assembly (NEB). Synonymous mutations were introduced in repair templates to prevent Cas9 re-cutting.

The CRISPR Design Tool was used to design guide RNAs targeting RelA and IKBKG (previously at http://crispr.mit.edu). Oligonucleotides were cloned into pSpCas9n (BB)-2A-Puro (PX462), which encodes Cas9 D10A nickase. The designed oligonucleotide pairs are as follows; IKBKG Sg1 (top) 5′-CACCGGCAGCAGATCAGGACGTAC-3′, IKBKG Sg1 (bottom) 5′-AAACGTACGTCCTGATCTGCTGCC-3′; and IKBKG Sg2 (top) 5′-CACCGCTGCACCATCTCACACAGT-3′, IKBKG Sg2 (bottom) 5′-AAACACTGTGTGAGATGGTGCAGC-3′.

A549 cells (ATCC) plated 48 h prior at 2–3×10^5^ cells per well in 6-well plates were transfected with linearized PX462-gRNA and donor plasmids using FuGENE HD (Promega; 3.5:1 reagent:DNA, total 3–4 μg). Following two weeks of transfection, cells were single-cell sorted into 96-well plates using Beckman Coulter MoFlo Astrios High Speed. Clones were isolated and screened by western blot and confirmed with live-cell imaging.

#### Western blot analysis

After 24 h in complete growth medium, A549 parental and EGFP-NEMO knock-in cells were treated then lysed at 4°C for 30 min in sodium dodecyl sulfate (SDS)-based lysis buffer (120 mM Tris-Cl, pH 6.8, 4% SDS) supplemented with protease and phosphatase inhibitors. Lysates were clarified by centrifugation at 12,000 × g for 10 min at 4°C, and protein concentrations were determined using a BCA assay (Pierce). Using SDS-polyacrylamide gel electrophoresis, 25 μg total protein per lane were separated and transferred to polyvinylidene difluoride membranes. Membranes were blocked in 5% milk in TBS for 1 h and incubated overnight at 4°C with primary antibodies against IKKγ (sc-8330, 1:1000) prepared in 5% milk in TBS-T. Blots were then incubated with LI-COR-conjugated secondary antibodies (1:10,000 dilution) and imaged using an Odyssey scanner to quantify band intensities.

#### Recombinant cytokines

Recombinant IL-1β proteins were obtained from multiple commercial sources for cross-species stimulation assays. Human, mouse, and rat IL-1β recombinant proteins were purchased from ThermoFisher (PeproTech brand; catalog numbers: 200–01B-2UG, 211–11B-2UG, and 400–01B-2UG, respectively). Bovine IL-1β was sourced from ThermoFisher (Invitrogen; RBOIL1BI). Feline and rabbit IL-1β/IL-1F2 proteins were acquired from R&D Systems (catalog numbers: 1796-FL-010 and 7464-RB-010, respectively). All cytokines were reconstituted and aliquoted according to the manufacturers’ instructions and stored at −80°C until use.

#### Live-cell imaging

Live-cell imaging was performed in an environmentally controlled chamber (37°C, 5% CO_2_) using a DeltaVision Elite microscope (GE Healthcare) equipped with a pco.edge sCMOS camera and an Insight solid-state illumination module. U2OS cells expressing fluorescent protein–tagged RelA were seeded at a density of 8,000 cells per well in no. 1.5 glass-bottom 96-well imaging plates (Matriplate) 24 h prior to imaging. One hour before acquisition, culture medium was replaced with phenol red–free FluoBrite DMEM (Gibco, A18967–01) supplemented with 10% fetal bovine serum (FBS; Corning), 100 U/mL penicillin, 100 μg/mL streptomycin, and 0.2 mM L-glutamine (Invitrogen).

EGFP-NEMO images were acquired every 3 min for 6 h using FITC filter sets and a 60× LUCPLFLN oil immersion objective (Video S1). Z-stacks were collected with eight planes at 0.5 μm intervals using a 0.04-s exposure time and 32% transmission. To image single-complex dynamics, EGFP-NEMO images were acquired every 10 s for 1 h using the same settings as above (Video S2). For NEMO spot tracking experiments, cells were stimulated with the indicated concentrations of IL-1 immediately before imaging. For imaging single-complex dynamics, a concentration of 10 ng/mL was used for IL-1β from human and feline, while a concentration of 500 ng/mL was used for bovine.

#### Quantification of EGFP-NEMO spots

EGFP-NEMO puncta were detected and quantified using dNEMO, a custom computational tool optimized for analyzing fluorescent puncta in fixed and live-cell time-lapse images.^40,61^ A detection threshold ranging from 1.5 to 2.8 was applied uniformly across all datasets. Puncta were considered valid only if they appeared in at least two contiguous slices within the 3D image stack (eight slices total). Pixel intensities for each punctum were background-corrected by averaging values from an annular ring (1-pixel width and offset) surrounding the spot. Single cells were manually segmented using dNEMO’s keyframing function. Spot features were extracted for each newly formed punctum following stimulation, yielding time-resolved, single-cell measurements.

#### Surface receptor quantification

Quantification of receptor numbers was performed as previously described in Cruz et al.,^40^ using fluorescence-activated cell sorting (FACS) with phycoerythrin (PE)-conjugated beads (BD Biosciences) and PE-conjugated IL-1R1, IL-1R2, IL-1R3, goat immunoglobulin G (IgG) and mouse IgG1 (R&D Systems). Cells were plated in six-well plates at a density of 10,000 to 20,000 cells per well and allowed to grow for forty-eight hours prior to staining. Cells were detached from plates using 2 mM EDTA in phosphate-buffered saline (PBS). Fc receptors were blocked using human BD Fc block antibody (BD Biosciences) for 10–15 min in darkness. Cells were then washed with 2% FBS in PBS and incubated with primary antibodies for 1 h at 4°C in darkness. Gating and calculation of molecules of equivalent soluble fluorochrome (MESF) were performed using FlowJo software (10.6.1_CL) and flowCore (2.16.0), flowWorkspace (4.16.0), CytoML (2.16.0), and openCyto (2.16.1) in R. Samples were corrected for background fluorescence by subtracting the mean fluorescence intensity (MFI) from samples stained with PE-conjugated isotype controls.

#### Thermal shift assay

IL-1 receptor-ligand binding affinities were measured by fluorescence-based thermal shift using a QuantStudio 3 system (Applied Biosystems). Purified, carrier-free recombinant human IL-1R1 (R&D Systems) and IL-1β orthologues (Invitrogen, R&D Systems) were resuspended in 50% (v/v) glycerol in distilled H2O for a final glycerol concentration of 12%. Reactions were prepared in 96-well plates (Applied Biosystems) with a total volume of 10 μL, containing 3 μM receptor, 3 μM ligand, 40X SYPRO Orange (Invitrogen), in 50 mM HEPES (Sigma-Aldrich) and 150 mM NaCl (Fisher Scientific) buffer. Plates were sealed with optical film and centrifuged to ensure collection and mixing at bottom of wells. Reactions were then held at 25°C for 2 min, increased 0.05°C per second until 99°C, then held at 99°C for 2 min. Fluorescence readouts were collected continuously throughout the melt protocol using the X4-M4 excitation-emission filter. Raw fluorescence values were normalized between 0 and 1 and smoothed using a quadratic Savitsky-Golay filter with a rolling window length of 15 temperature points. The maximum derivative of the melt curve was determined as the melt temperature.

#### Single particle tracking of EGFP-NEMO spots

EGFP-NEMO spot location and intensity data obtained from dNEMO were used for single-spot tracking with the *uTrack* package in MATLAB.^62,68^ Tracking parameters were optimized to improve performance for IL-1–induced puncta. Specifically, the maximum allowed gap between detections was reduced from five to three frames, and the gap-closing penalty was lowered from 1.5 to 1. The minimum track segment length used for gap closing was increased from one to three frames. Spot properties, including intensity and size, were associated with their corresponding trajectories to compute integrated intensity over time. Only spots that formed within the first four minutes of imaging were included in the analysis.

#### Extracting features from spot trajectories

Quantitative descriptors of EGFP-NEMO trajectories were extracted using custom MATLAB and Python scripts from either single-cell or single-complex data. For long time-course experiments, trajectories were generated based on the number of NEMO puncta per cell over time. For short-term tracking experiments, trajectories reflected the integrated intensity of all NEMO spots within a cell at each time point. The following metrics were computed for each trajectory.

1. Area under the curve (AUC): Total integrated value of the trajectory, representing either the cumulative number of NEMO spots or the total spot intensity per cell.
2. Maximum value (Max): Peak number of NEMO spots or peak integrated intensity.
3. Rate of rise (*Rate*_*up*_): Maximum slope (absolute value) calculated from a linear fit to three consecutive time points between time zero and the time of maximum signal.
4. Rate of decay (*Rate*_*down*_): Maximum slope (absolute value) from a linear fit to three time points between the time of maximum signal and the end of the trajectory.
5. Time of maximum (*t*_*max*_): Time point at which the trajectory reaches its maximum value.
6. Time to half-maximum rise (*t*_50,*up*_): Time at which the trajectory first reaches 50% of its maximum value.
7. Time to half-maximum decay (*t*_50,*down*_): Time at which the trajectory falls back to 50% of its maximum value.
8. Full width at half maximum (FWHM): Duration between *t*_50,*up*_ and *t*_50,*down*_ indicating the width of the response at half-maximum intensity.
9. Adaptation time: Time at which the trajectory first reaches its lower plateau value.

#### Sigmoidal fitting of AUC and max features

Sigmoidal curve fitting of extracted AUC and Max features from NEMO spot number trajectories was performed using a custom Python script. For each species and cytokine concentration, the mean feature value was calculated and used as the input for curve fitting. A standard sigmoid equation was applied:

$$y=\frac{y_{MAX}}{1+\frac{{K_{A}}^{n}}{X}}$$

Fits were computed using the curve_fit function from the *SciPy* package. Estimated parameter values and corresponding R^2^ scores are summarized in Table S1.

#### Quantifying noise in feature distributions

To evaluate cell-to-cell variability in extracted features from NEMO spot number trajectories, we computed the Fano factor, a common measure of noise defined as the variance-to-mean ratio. For each species and cytokine concentration, we extracted single-cell values for five dynamic descriptors: AUC, Max, T_MAX_, FWHM, and Adaptation Time. Using custom Python scripts, we first grouped single cell feature values by species and stimulation dose. For each group, we calculated the mean and variance, then computed the Fano factor as:

$$\mathrm{fano}\;\mathrm{factor}\;=\;\frac{\mathrm{variance}}{\mathrm{mean}}$$

Values were excluded if the group mean was zero or undefined. To visualize how noise varied across species and concentrations, we generated two complementary plots per feature: (1) a line plot of the Fano factor across cytokine concentrations for each species, and (2) a species-wise boxplot showing the distribution of Fano values across all doses.

This analysis allowed us to quantify the extent of single-cell heterogeneity for each extracted feature, across both species and stimulation conditions.

#### Simulation of CI-like complex formation

We modeled the bulk assembly of CI-like complexes using the Gillespie algorithm to determine the range in relative IL-1β–IL-1R1 affinity necessary to recapitulate experimental measurements of EGFP-NEMO puncta numbers and timing. The key variable simulated was the number *N* of mature CI-like complexes resulting from the assembly cascade previously described. The relative binding affinity *Aff* altered only the on-rate *k*_1,*on*_ and off-rate *k*_1,*off*_, for which the values were scaled up or down, respectively, by a factor of *Aff*. All other parameters were held constant. The parameter values are described in Table S1.

#### Sigmoidal fitting of Gillespie simulations

Sigmoidal curve fitting of extracted AUC and Max features from stochastically simulated NEMO spot number trajectories was performed using a custom Python script. For each relative affinity and cytokine concentration, the mean feature value was calculated and used as the input for curve fitting. A standard sigmoid equation was applied:

$$y=\frac{y_{MAX}}{1+e^{-K_{A}\left(x-x_{0}\right)}}$$

Fits were computed using the curve_fit function from the *SciPy* package. Estimated parameter values and corresponding R^2^ scores are summarized in Table S1.

#### Single-complex contact duration modeling

We modeled the intensity dynamics of individual EGFP-NEMO puncta using a hybrid deterministic–stochastic (HyDeS) framework to test how ligand–receptor contact duration influences the assembly of signalosome-like structures. Two key variables were simulated: the intensity *I* of each NEMO spot, representing a continuous approximation of ubiquitin chain size and NEMO activity at that site; and a feedback variable $X_{\mathrm{basal}}$, which serves as a proxy for the influence of basal deubiquitinating enzyme (DUB) activity present in resting cells. Basal feedback was modeled to increase proportionally with spot intensity, representing DUB recruitment to active complexes.

The model comprises *2N* coupled ordinary differential equations (ODEs) for *N* NEMO spots: one equation for the intensity $I_{i}$ of each spot and one for the corresponding feedback variable $X_{\mathrm{basali}}$. Simulations were performed with 75 individual spots forming at regular intervals, approximating the response of a single cell following cytokine stimulation.

$$\begin{array}{c} \frac{d\left[I_{i}\right]}{dt}=P_{\mathrm{form}}\times P_{\mathrm{bound}}\left(\frac{K_{\mathrm{growth}}}{K_{\mathrm{limit}}}+\left[I_{i}\right]\right)-Kd_{\mathrm{basal}}\times X_{\mathrm{basali}}\times \left[I_{i}\right]\\ \frac{d\left[X_{\mathrm{basal}i}\right]}{dt}=Kx_{\mathrm{basal}}\times \left[I_{i}\right]-Kxd_{\mathrm{basal}}\times \left[X_{\mathrm{basal}i}\right] \end{array}$$

The variables and parameter values used in the model are as described in Table S1. We examined the effects of contact duration on the single complex dynamics in three different ways: (1) toggling complex formation on and off based on binding state, corresponding to lower $P_{\mathrm{bound}}$ for lower affinities; (2) modulating the rate of complex formation upon binding, corresponding to lower $K_{\mathrm{growth}}$ for lower affinities; and (3) suppressing DUB activity at higher affinities. For the latter, $Kd_{\mathrm{basal}}$ is divided by the affinity, which reflects DUB inhibition due to higher affinities, in the following way:

$$Kd_{\mathrm{basal}}^{\prime }=\frac{500\times Kd_{\mathrm{basal}}}{500\times \mathrm{affinity}+1}$$

An arbitrary multiplier was used to scale up the effects of affinity on $Kd_{\mathrm{basal}}$. The descriptions and default values for all parameters are listed in Table S1.

#### Distance tree construction

Multiple sequence alignment via MAFFT was performed on recombinant IL-1β sequences from each species.^69^ Human IL-1β binding regions were annotated using a distance threshold of 4.5Å between residues of IL-1β to IL-1R1 or IL-1R3 in the complex’s crystal structure (PDB: 4DEP). Unweighted Pair-Group Method using Arithmetic averages trees were constructed with BLOSUM62 similarity for both the full sequence and annotated binding region.

#### Chimeric structure prediction

Predicted structures for each IL-1β–IL-1R1 and IL-1β–IL-1R1–IL-1R3 complex were generated using the Alphafold3 server, which was provided the recombinant species-specific IL-1β sequence and the human IL-1R1 or human IL-1R1 and human IL-1R3 sequence(s). All five highest-pLDDT predictions were considered as optimal binding candidates.

#### Molecular dynamics system preparation

The crystal structure for the human IL-1β–IL-1R1 complex (PDB: 1ITB) was downloaded, prepared for use in the AMBER suite with PDBfixer v. 1.11, parameterized with the AMBER ff15IPQ force field,^70^ and solvated with a 12Å TIP3P water box with 12Å padding.

For each bipartite or tripartite chimeric system, Open Babel v. 3.1.0^65^ was used to convert AF3 structures to a complexed IL-1β–IL-1R1 or IL-1β–IL-1R1–IL-1R3, isolated IL-1β or IL-1R3, and isolated IL-1R1 or IL-1β–IL-1R1 PDB file for each of the 5 predicted structures. Each was then prepared for use in the AMBER suite via pdb4amber v. 1.6.dev.^63^ and parameterized with the AMBER ff15IPQ force field.^70^ An additional neutralized and 12Å TIP3P solvated structure file was produced for each complex.

#### Molecular dynamics simulation

Classical MD simulations for each solvated complex were performed with the Amber 24 software package^63^ with a 2-fs time step. Water and ions were first minimized for 50ps with a protein restraint weight of 2.0 kCal/mol Å^2^. Under the same protein restraint, each system was heated with constant volume from 100K to 298K over 50 ps, followed by 50 ps of constant pressure relaxation. Each system was then equilibrated under no restraint for 1 ns. Finally, five successive 5ns production simulations for each system with no restraint were performed. Velocities were reset between each simulation.

#### Free energy estimation

100 frames from each of the five 5ns simulations were used in MM/GBSA^45^ to estimate the total binding free energy. The lowest MM/GBSA binding free energy simulation was chosen as the ideal conformation for per-residue decomposition of binding free energy using MM/PBSA^45^ and structural comparison.

#### C-terminal distance measurement

The C-terminal distance was characterized across every frame of the five 5ns simulations for a given orthologue’s lowest ΔG° tripartite complex. cpptraj version 6.18.1^67^ was used to measure the distance between C-terminal alpha carbons of IL-1R1 and IL-1R3.

### QUANTIFICATION AND STATISTICAL ANALYSIS

To account for the low number of EGFP-NEMO spots detected in U2OS cells stimulated with 500 ng/mL Bovine IL-1β, bootstrapping was performed to enable statistical comparisons across conditions. For each IL-1β species condition (Human, Feline, and Bovine), we resampled the set of tracked EGFP-NEMO trajectories 100,000 times using a sample size of 5 per iteration. Resampling was implemented using a custom MATLAB script. As described in Figure 4F legend, differences between groups of single complex trajectories were assessed using left-tailed t-tests. Box-plots show Q1, median, and Q3, with whiskers denoting 1.5x IQR (interquartile range).

## Supplementary Material

1

2

3

4

5

6

SUPPLEMENTAL INFORMATION

Supplemental information can be found online at https://doi.org/10.1016/j.celrep.2025.116637.

## Figures and Tables

**KEY RESOURCES TABLE:** 

REAGENT or RESOURCE	SOURCE	IDENTIFIER
Antibodies		
IL-1R1 PE-Conjugated Antibody	R&D Systems	Cat# FAB269P; RRID: AB_2124912
IL-1R2 PE-Conjugated Antibody	R&D Systems	Cat# FAB663P; RRID: AB_1964613
IL-1R3 PE-Conjugated Antibody	R&D Systems	Cat# FAB676P; RRID: AB_10717521
Goat IgG PE-Conjugated Antibody	R&D Systems	Cat# IC108P; RRID: AB_10174792
Mouse IgG1 PE-Conjugated Antibody	R&D Systems	Cat# IC002P; RRID: AB_357242
Human BD Fc Block	BD Biosciences	Cat# 564219; RRID: AB_2728082
IKKγ Antibody	Santa Cruz	Cat# sc-8330; RRID: AB_2124846
β-Actin Antibody	Cell Signaling Technology	Cat# 3700; RRID: AB_2242334
IRDye 680RD Anti-Rabbit	LICOR	Cat# 926-68071; RRID: AB_10956166
IRDye 800CW Anti-Mouse	LICOR	Cat# 925-32210; RRID: AB_2687825
Chemicals, peptides, and recombinant proteins		
Human IL-1B	PeproTech	Cat# 200-01B
Feline IL-1B	R&D Systems	Cat# 1796-FL
Feline IL-1B (Carrier Free)	R&D Systems	Cat# 1796-FL/CF
Rat IL-1B	PeproTech	Cat# 400-01B
Mouse IL-1B	PeproTech	Cat# 211-11B
Rabbit IL-1B	R&D Systems	Cat# 7464-RB
Bovine IL-1B	Invitrogen	Cat# RBOIL1BI
Human IL-1 RI	R&D Systems	Cat# 269-1R/CF
SYPRO Orange Protein Gel Stain	Invitrogen	Cat# S6651
Fluorobrite DMEM	Gibco	Cat# A18967-01
Trypsin	Corning	Cat# 25-053-CI
F-12K	Gibco	Cat# 21127-022
McCoy’s	Gibco	Cat# 16600-082
PBS	Cytiva	Cat# SH30256.01
FBS	Corning	Cat# 35-010-CV; Lot# 03322001
Penicillin/Streptomycin	Corning	Cat# 30-002-CI
L-Glu	Corning	Cat# 25-005-CI
Glycerol	Fisher Scientific	Cat# G33
dH2O	Invitrogen	Cat# 10977015
HEPES	Sigma-Aldrich	Cat# H4034
NaCl	Fisher Scientific	Cat# S271
AatII	New England Biolabs	Cat# R0117
Protease Inhibitor	Roche	Cat# 11836170001
Phosphatase Inhibitor	Thermo Scientific	Cat# A32957
Critical commercial assays		
BD Quantibrite Beads	BD Biosciences	Cat# 340495; Lot #67984
MicroAmp EnduraPlate Optical 96-Well Clear Reaction Plate	Applied Biosystems	Cat# 4483354
Optical Adhesive Covers	Applied Biosystems	Cat# 4360954
FuGENE HD Transfection Reagent	Promega	Cat# E2311
Pierce BCA Protein Assay Kit	Thermo Scientific	Cat# 23225
PlasmoTest Mycoplasma Detection Kit	InvivoGen	Cat# rep-pt1
Deposited data		
Crystal Structure, IL1B Signaling Complex	Thomas et al.^46^	PDB: 4DEP
Crystal Structure, IL1R1 In Complex with IL1B	Vigers et al.^42^	PDB: 1ITB
Experimental models: Cell lines		
U2OS	ATCC	RRID: CVCL_0042
A549	ATCC	RRID: CVCL_0023
Oligonucleotides		
IKBKG-eGFP Primers: Forward: 5′-TCTGCTGGGTAAGGATGTG-3′ Reverse: 5′-GCTCTTGATTCTCCTCCAGGCAG-3′	Pabon et al.^58^	N/A
IKBKG sgRNA 1: Top: 5′-CACCGGCAGCAGATCAGGACGTAC-3′ Bottom: 5′-AAACGTACGTCCTGATCTGCTGCC-3′	Pabon et al.^58^	N/A
IKBKG sgRNA 2: Top: 5′-CACCGCTGCACCATCTCACACAGT-3′ Bottom: 5′-AAACACTGTGTGAGATGGTGCAGC-3′	Pabon et al.^58^	N/A
RelA sgRNA 1 Top: 5′-CACCGCTCGTCTGTAGTGCACGCCG-3′ Bottom: 5′-AAACCGGCGTGCACTACAGACGAGC-3′	Pabon et al.^58^	N/A
RelA sgRNA 2 Top: 5′-CACCGAGAGGCGGAAATGCGCCGCC-3′ Bottom: 5′-AAACCGCGGCGCATTTCCGCCTCTC-3′	Pabon et al.^58^	N/A
Recombinant DNA		
pSpCas9n(BB)-2A-Puro (PX462)	Ran et al.^59^	RRID: Addgene_48141
Software and algorithms		
ImageJ	Schneider et al.^60^	https://imagej.net/ij/
dNEMO (1.1.0)	Kowalczyk et al.^61^	https://github.com/recleelab/dNEMO-1.1.0
MATLAB (R2018b)	MathWorks	https://www.mathworks.com/products/matlab.html
AlphaFold3 Server	Abramson et al.^8^	https://alphafoldserver.com
uTrack (2.5)	Jaqaman et al.^62^	https://github.com/DanuserLab/u-track
AMBER 22	Case et al.^63^	https://ambermd.org/InstSingularity.php
AMBER 24	Case et al.^64^	https://ambermd.org/GetAmber.php
PyMOL	Schrödinger LLC	https://github.com/schrodinger/pymol-open-source
OpenBabel (3.1.0)	O’Boyle et al.^65^	https://sourceforge.net/projects/openbabel/
Python (3.13.2, 3.12.2, 2.7)	Python	https://www.python.org/
R (4.4.1)	R Core Team	https://www.r-project.org/
FlowJo (10.6.1_CL)	BD Biosciences	https://www.flowjo.com/
MAFFT (7)	Katoh et al.^66^	https://mafft.cbrc.jp/alignment/software/
cpptraj (6.18.1)	Roe et al.^67^	https://ambermd.org/InstSingularity.php
Adobe Illustrator (2025)	Adobe Inc.	https://www.adobe.com/products/illustrator.html
Original code for simulations	This paper	https://doi.org/10.5281/zenodo.17353771

## Highlights

- Mammalian IL-1β orthologs bind human IL-1 receptors with varying affinities
- Affinity of IL-1β orthologs determines probability of forming receptor complexes
- IL-1β complexes signal by a quantized packet-based mechanism
- Signaling output from single activated receptor complexes is affinity independent
