# Supplementary material for: NEMO recruitment at single cytokine-receptor complexes shows quantized dynamics independent of ligand affinity
Source: Cell Rep. Author manuscript; Available in PMC 2026 Jan 24. (PMC12831165; doi:10.1016/j.celrep.2025.116637)
Supplement: 1 [file NIHMS2132741-supplement-1.pdf]

Cell Reports, Volume *44*

Supplemental information

NEMO recruitment at single cytokine-receptor complexes shows quantized dynamics independent of ligand affinity

A. Hyun Kim, Benjamin Krummenacher, Jason Yeung, David R. Koes, and Robin E.C. Lee

Figure S1: Comparative Structural and Energetic Analysis of IL-1β–IL-1R1 Interactions Across Species, Related to Figure 1

A. Multiple sequence alignment of IL-1β sequences from a panel of 12 species. Predicted residues involved in IL-1R1/IL-1R3 binding denoted by red rectangles.

B. Differences between the crystal structure and predicted structure for the IL-1β–IL-1R1 and IL-1β–IL-1R1–IL-1R3 complexes in terms of predicted binding conformations and contributions to changes in free energy resulting from binding.

C. Estimated changes in binding free energy of generated IL-1β/IL-1R1 and IL-1β/IL-1R1/IL-1R3 complexes relative to that of the human crystal structure.

D. Distributions of distances throughout MD simulation between C termini of IL-1R1 and IL-1R3 in the tripartite complexes. C-termini IL-1R1–IL-1R3 distance found in the experimental structure shown in red.

Figure S1

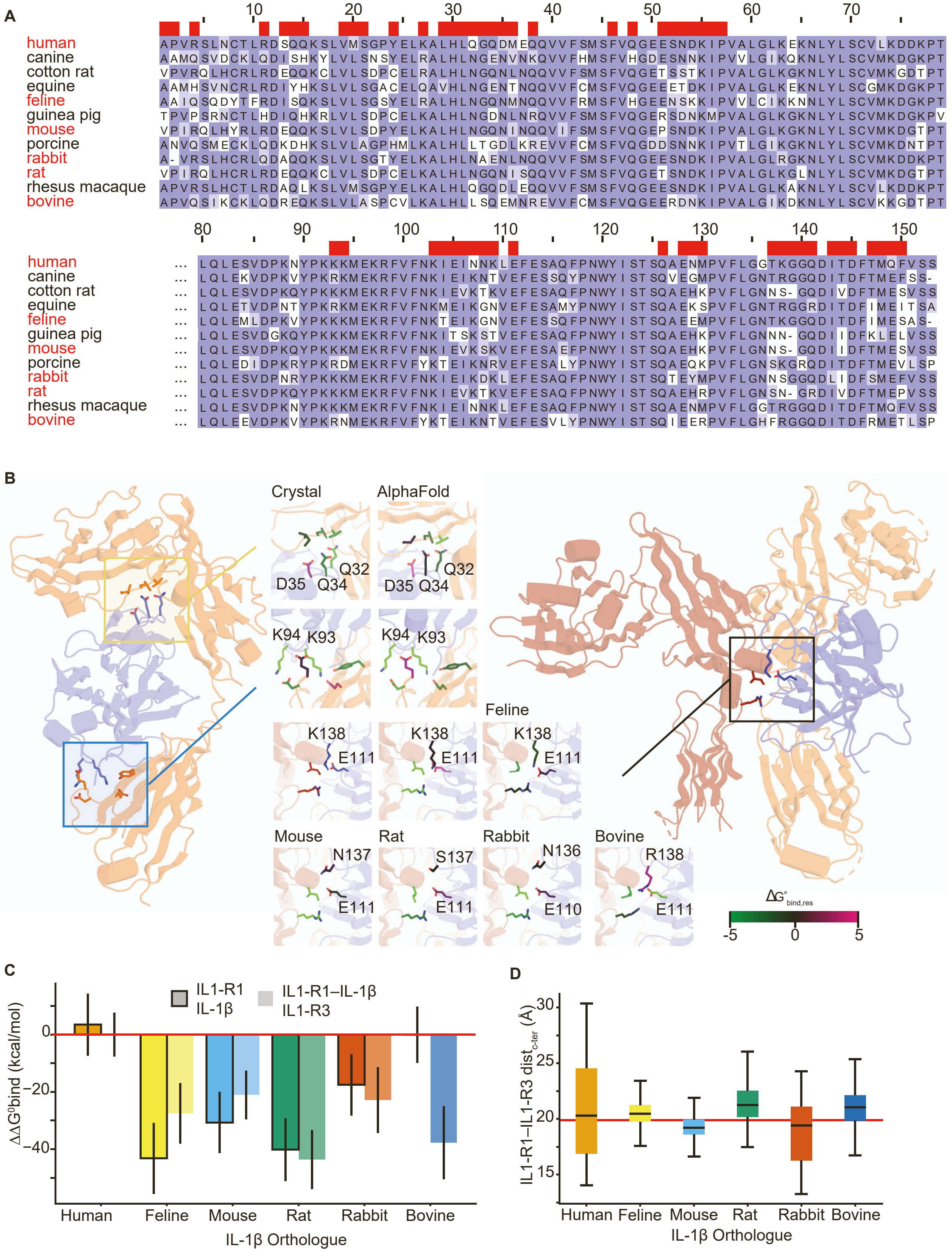
A
10 20 30 40 50 60 70
human APVRSLNCTLRDSQQKSLVMSGPYELKALHLQGQDMEQQVVFSMSFVQGEESNDKIPVALGLKEKNLYLSCVLKDDKPT
canine AAMQSVDCKLQDISHKYLVLSNSYELRALHLNGENVNKQVVFHMSFVHGDESNNKIPVVLGIKQKNLYLSCVMKDGKPT
cotton rat VPVRQLHCRLRDEQQKCLVLSDPCELRALHLNGQNLNQQVVFSMSFVQGETSSTKIPVALGLKGKNLYLSCVMKGDTPT
equine AAMHSVNCRLRDIYHKSLVLSGACELQAVHLNGENTNQQVVFCMSFVQGEETDKIPVALGLKEKNLYLSCGMKDGKPT
feline AAIQSQDYTFRDISQKSLVLSGSYELRALHLNGQNMNQQVVFRMSFVHGEENSKKIPVVLCIKKNNLYLSCVMKDGKPT
guinea pig TPVPSRNCTLHDIQHKRLVLSDPCELKALHLNGDNLNRQVVFSMSFVQGERSDNKMPVALGLKGKNLYLSCVMKDGKPV
mouse VPIRQLHYRLRDEQQKSLVLSDPYELKALHLNGQNINQQVIFSMSFVQGEPSNDKIPVALGLKGKNLYLSCVMKDGTPT
porcine ANVQSMECKLQDKDHKSLVLAGPHMLKALHLLTGDLKREVVFCMSFVQGDDSNNKIPVTLGIKGKNLYLSCVMKDNTPT
rabbit A-VRSLHCRLQDAQQKSLVLSGTYELKALHLNAENLNQQVVFSMSFVQGEESNDKIPVALGLRGKNLYLSCVMKDDKPT
rat VPIRQLHCRLRDEQQKCLVLSDPCELKALHLNGQNISQQVVFSMSFVQGETSNDKIPVALGLKGLNLYLSCVMKDGTPT
rhesus macaque APVRSLHCTLRDAQLKSLVMSGPYELKALHLQGQDLEQQVVFSMSFVQGEESNDKIPVALGLKAKNLYLSCVLKDDKPT
bovine APVQSIKCKLQDREQKSLVLASPCVLKALHLLSQEMNREVVFCMSFVQGEERDNKIPVALGIKDKNLYLSCVKKGDTPT
80 90 100 110 120 130 140 150
human … LQLESVDPKNYPKKKMEKRFVFNKIEINNKLEFESAQFPNWYISTSQAENMPVFLGGTKGGQDITDFTMQFVSS
canine … LQLEKVDPKVYPKRKMEKRFVFNKIEIKNTVEFESSQYPNWYISTSQVEGMPVFLGNTRGGQDITDFTMEFSS-
cotton rat … LQLESVDPKQYPKKKMEKRFVFNKIEVKTKVEFESAQFPNWYISTSQAEHKPVFLGNNS-GQDIVDFTMESVSS
equine … LQLETVDPNTYPKRKMEKRFVFNKMEIKGNVEFESAMYPNWYISTSQAEKSPVFLGNTRGGRDITDFIMEITSA
feline … LQLEMLDPKVYPKKKMEKRFVFNKTEIKGNVEFESSQFPNWYISTSQAEEMPVFLGNTKGGQDITDFIMESAS-
guinea pig … LQLESVDGKQYPKKKMEKRFVFNKITSKSTVEFESAQFPNWYISTSQAEHKPVFLGNNN-GQDIIDFKLELVSS
mouse … LQLESVDPKQYPKKKMEKRFVFNKIEVKSKVEFESAEFPNWYISTSQAEHKPVFLGNNS-GQDIIDFTMESVSS
porcine … LQLEDIDPKRYPKRDMEKRFVFYKTEIKNRVEFESALYPNWYISTSQAEQKPVFLGNSKGRQDITDFTMEVLSP
rabbit … LQLESVDPNRYPKKKMEKRFVFNKIEIKDKLEFESAQFPNWYISTSQTEYMPVFLGNNSGGQDLIDFSMEFVSS
rat … LQLESVDPKQYPKKKMEKRFVFNKIEVKTKVEFESAQFPNWYISTSQAEHRPVFLGSN-GRDIVDFTMEPVSS
rhesus macaque … LQLESVDPKNYPKKKMEKRFVFNKIEINNKLEFESAQFPNWYISTSQAENMPVFLGGTRGGQDITDFTMQFVSS
bovine … LQLEEVDPKVYPKRNMEKRFVFYKTEIKNTVEFESVLYPNWYISTSQIEERPVFLGHFRGGQDITDFRMETLSP
B
Crystal
AlphaFold
Q32
D35 Q34
K94 K93
Feline
K138
E111
Mouse
Rat
Rabbit
Bovine
N137
S137
N136
R138
E110
ΔG°bind,res
-5
0
5
C
IL1-R1 IL-1β
IL1-R1–IL-1β IL1-R3
ΔΔG0bind (kcal/mol)
0
-20
-40
Human
Feline
Mouse
Rat
Rabbit
Bovine
IL-1β Orthologue
D
IL1-R1–IL1-R3 dist c-ter (Å)
30
25
20
15
Human Feline Mouse Rat Rabbit Bovine
IL-1β Orthologue

Figure S2: Cross-Species and Cross-Cell Line Comparison of EGFP-NEMO Complex Formation and Intensity Profiles in Response to IL-1β Stimulation, Related to Figure 2

A. Time-lapse images of EGFP-NEMO in live U2OS cells stimulated with 250 ng/mL IL-1β from the indicated species. Line scans were performed at 24 minutes post-stimulation (blue lines). Scale bar, 20 μm.

B. Line intensity profiles of two representative EGFP-NEMO spots corresponding to line scans shown in (A), from cells stimulated with 250 ng/mL IL-1β from the indicated species.

C. Western blot of NEMO expression in stock (WT) and A549 cells that were CRISPR-modified to express EGFP-NEMO. The molecular weight of NEMO in the CRISPR-modified cells is upward shifted by 32 kDa, consistent with the expected endogenous fusion protein.

D. Single-cell time courses of EGFP-NEMO complex formation in A549 cells in response to indicated IL-1β concentrations and orthologues.

E. Quantibrite FACs analysis of surface IL-1R1, IL-1R2, and IL-1R3 expression in U2OS and A549 cells in indicated replicates.

F. Averaged surface receptor expression numbers in U2OS and A549, calculated from results in (E).

Figure S2

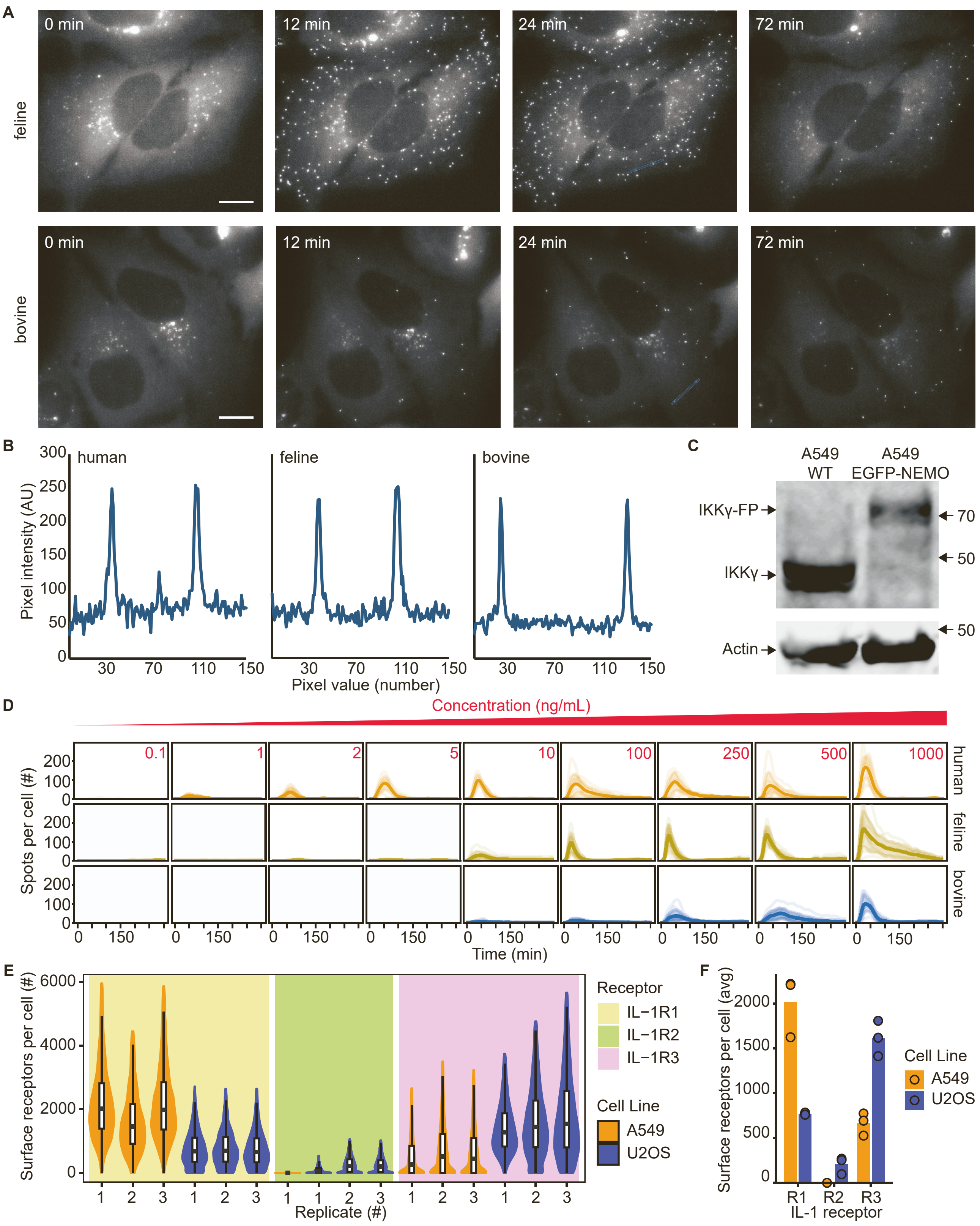

A
0 min
12 min
24 min
72 min
feline
bovine
B
human
feline
bovine
Pixel intensity (AU)
Pixel value (number)
C
A549 WT
A549 EGFP-NEMO
IKKγ-FP
IKKγ
Actin
70
50
50
D
Concentration (ng/mL)
0.1
1
2
5
10
100
250
500
1000
human
feline
bovine
Spots per cell (#)
Time (min)
E
Surface receptors per cell (#)
Replicate (#)
Receptor
IL−1R1
IL−1R2
IL−1R3
Cell Line
A549
U2OS
F
Surface receptors per cell (avg)
R1
R2
R3
IL-1 receptor
Cell Line
A549
U2OS

Figure S3: Experimental and Simulated Dose–Response Analysis of EGFP-NEMO Complex Formation Using the MAX Descriptor, Related to Figure 3

A. Sigmoid curves fitted to the mean maximum spot number (MAX) from experimental single-cell time courses across IL-1β concentrations and species, revealing species-specific dose–response relationships. The EC50, defined as the concentration at which 50% of the maximal response is reached, is indicated. See Table S1 for fit parameters.

B. EC50 values for each species derived from the MAX descriptor fits shown in (A), indicating the concentration at which 50% of the maximal response is reached.

C. Stochastic simulations of spot number time courses across species and concentrations, using affinity parameters optimized to match experimental data. Fitted affinity values are shown above each plot, representing relative signaling competency of each species. See also Table S1.

D. Simulated dose–response curves for the MAX descriptor, recapitulating relative IL-1β–IL-1R1 affinity-dependent complex formation dynamics. See also Table S1.

E. Simulated EC50 values derived from the MAX descriptor for a range of IL-1β–IL-1R1 affinities, reflecting predicted net affinity of IL-1β to form signaling competent complexes across species.

Figure S3

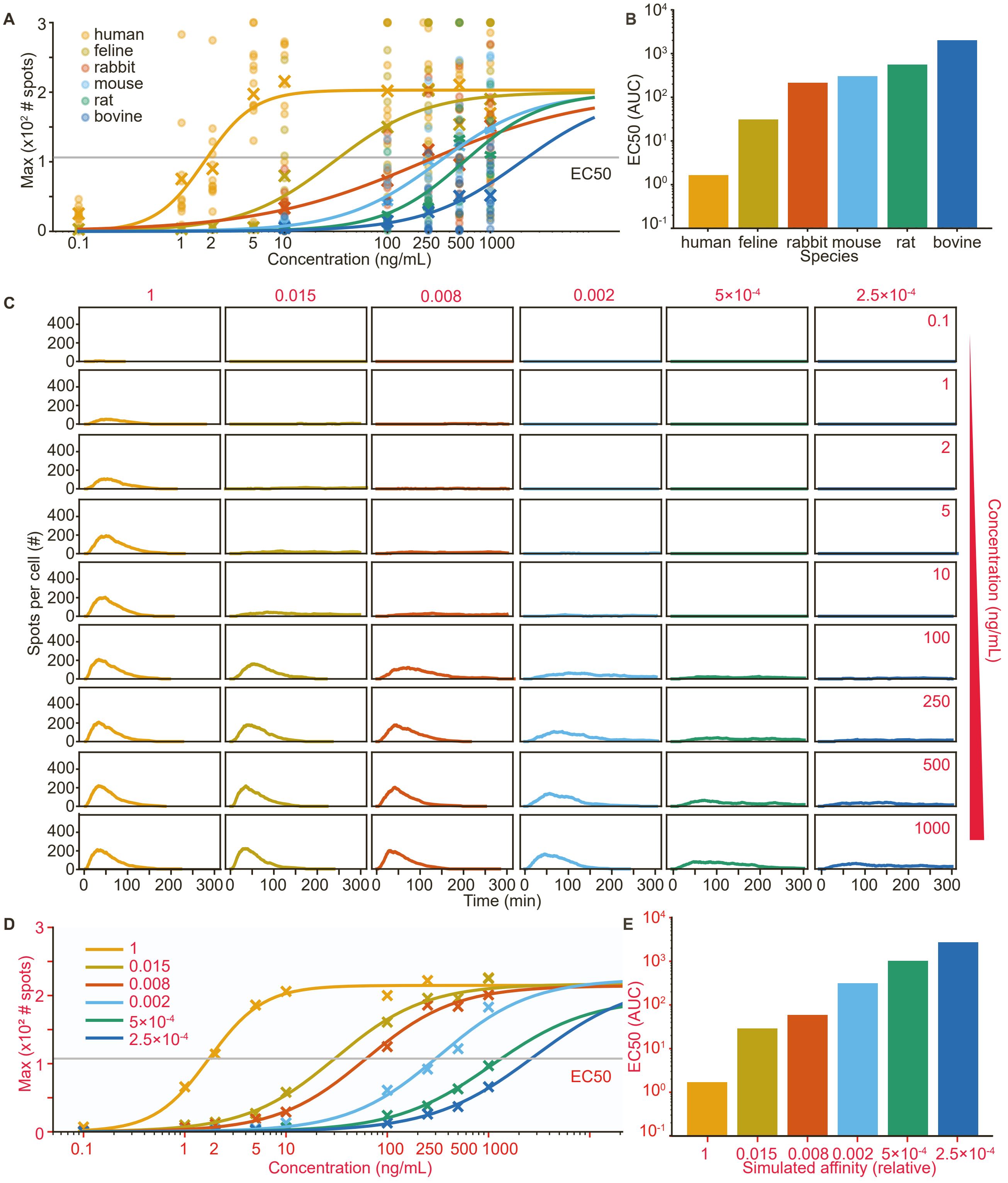

A
B
C
D
E
human
feline
rabbit
mouse
rat
bovine
Max (x10² # spots)
Concentration (ng/mL)
EC50
EC50 (AUC)
Species
Spots per cell (#)
Time (min)
Concentration (ng/mL)
1
0.015
0.008
0.002
5×10⁻⁴
2.5×10⁻⁴
Simulated affinity (relative)

Figure S4: Simulated Effects of Contact Duration on EGFP-NEMO Single-Complex Dynamics, Related to Figure 4

A. Conceptual schematic extending Figure 4A, illustrating three hypothetical mechanisms by which ligand–receptor contact duration may influence EGFP-NEMO complex dynamics following its assembly: (1) toggling complex formation on and off based on binding state; (2) modulating the rate of complex formation upon binding; and (3) suppressing DUB activity at higher affinities.

B. Stochastic simulations of single-complex spot intensity trajectories under each of the three mechanisms described in (A). See Table S1 for parameter values. Box plots summarize key trajectory descriptors, demonstrating how a one-order-of-magnitude reduction in receptor–ligand affinity alters single-complex dynamics.

Figure S4

A

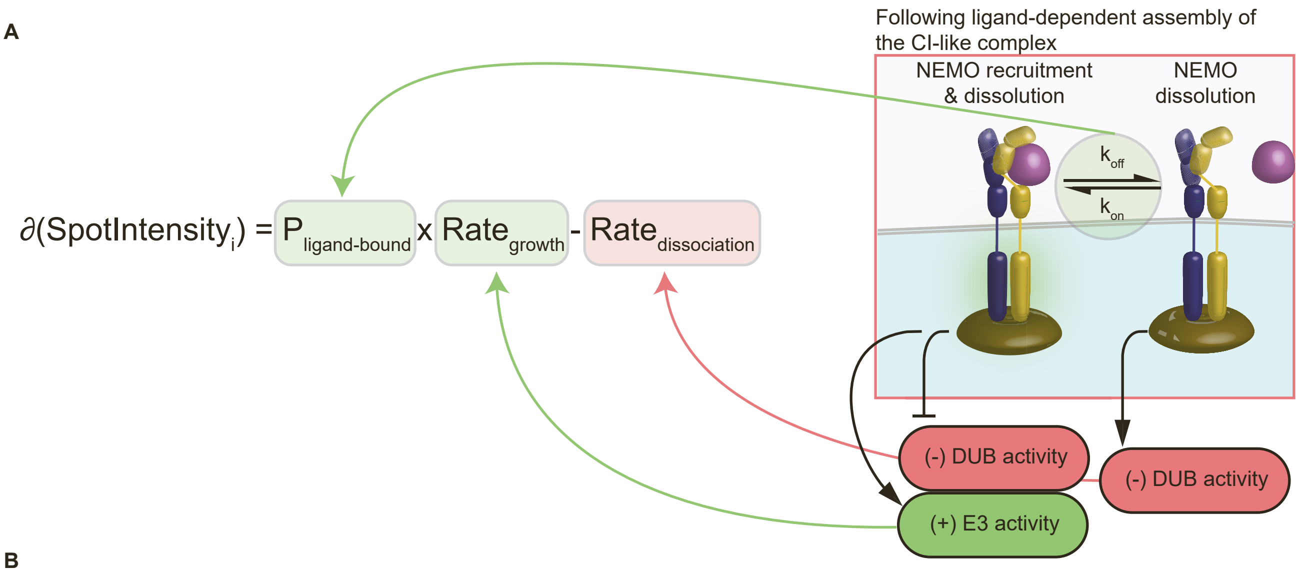
Following ligand-dependent assembly of the CI-like complex
NEMO recruitment & dissolution
NEMO dissolution
k_{off}
k_{on}
$\partial(SpotIntensity_i) = P_{ligand-bound} \times Rate_{growth} - Rate_{dissociation}$
(-) DUB activity
(-) DUB activity
(+) E3 activity

B

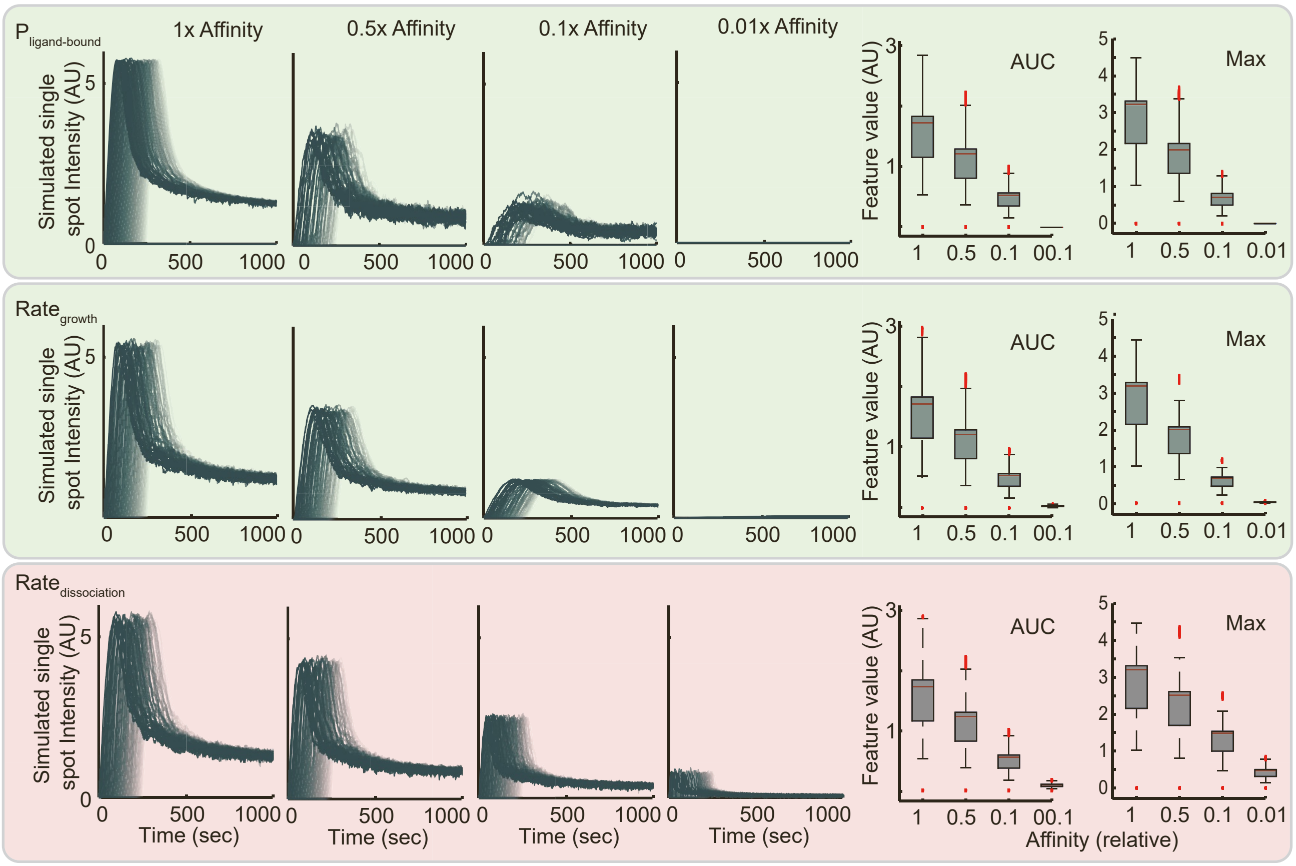
$P_{ligand-bound}$
$Rate_{growth}$
$Rate_{dissociation}$
1x Affinity
0.5x Affinity
0.1x Affinity
0.01x Affinity
Simulated single spot Intensity (AU)
Time (sec)
Feature value (AU)
AUC
Max
Affinity (relative)
